# Phosphate uptake by the phosphonate transport system PhnCDE

**DOI:** 10.1186/s12866-019-1445-3

**Published:** 2019-04-16

**Authors:** Raffaele Stasi, Henrique Iglesias Neves, Beny Spira

**Affiliations:** Departamento de Microbiologia, Instituto de Ciências Biomédicas Universidade de São Paulo, São Paulo-SP, Brazil

**Keywords:** Phosphonates, Phosphate, Phn, PHO regulon

## Abstract

**Background:**

Phosphate is a fundamental nutrient for all creatures. It is thus not surprising that a single bacterium carries different transport systems for this molecule, each usually operating under different environmental conditions. The phosphonate transport system of *E. coli* K-12 is cryptic due to an 8 bp insertion in the *phnE* ORF.

**Results:**

Here we report that an *E. coli* K-12 strain carrying the triple knockout *ΔpitA* *Δpst* *Δugp* reverted the *phnE* mutation when plated on complex medium containing phosphate as the main phosphorus source. It is also shown that PhnCDE takes up orthophosphate with transport kinetics compatible with that of the canonical transport system PitA and that Pi-uptake via PhnCDE is sufficient to enable bacterial growth. Ugp, a glycerol phosphate transporter, is unable to take up phosphate.

**Conclusions:**

The phosphonate transport system, which is normally cryptic in *E. coli* laboratory strains is activated upon selection in rich medium and takes up orthophosphate in the absence of the two canonical phosphate-uptake systems. Based on these findings, the PhnCDE system can be considered a genuine phosphate transport system.

**Electronic supplementary material:**

The online version of this article (10.1186/s12866-019-1445-3) contains supplementary material, which is available to authorized users.

## Background

Phosphorus is a macronutrient of utmost importance to all living beings. It is thus not surprising that bacteria developed several different mechanisms of phosphorus acquisition. Particularly, the PHO regulon, a set of genes involved in the acquisition and metabolism of phosphorus containing molecules in response to phosphate (Pi)-shortage in the medium is present across bacterial phyla. The PHO regulon of *E. coli* comprises more than 30 genes [1]. The most well characterized genes of the PHO regulon are *phoB-phoR*, the operons *pstSCAB-phoU*, *ugpBAEC*, *phnCDEFGHIJKLMNOP* and *phoA* that respectively encode the two-component system that controls the transcription of the regulon, an ABC-type Pi-transport system, an ABC-type glycerol phosphate transport system, a 14-gene operon involved in phosphonate (Pn) transport and assimilation and a periplasmic alkaline phosphatase (AP). Genes belonging to the PHO regulon are synchronously induced by Pi-shortage [2]. For the sake of simplicity, the operons *pstSCAB-phoU*, *ugpBAEC*, *phnCDEFGHIJKLMNOP* will be respectively shortened to *pst*, *ugp* and *phn*.

Most, but not all available phosphorus in nature is in its most oxidized state (+5), also known as phosphate. Phosphonates (P valence +3) are phosphorus-containing organic molecules in which the P atom is linked directly to C in a stable chemical bond. Though less common in nature than organic phosphates, many Pn molecules, such as the antibiotic fosfomycin and the herbicide glyphosate are of clinical and environmental importance [3, 4]. The first three genes of the *phn* operon - *phnCDE*, encode an ABC-type Pn transport system. The polypeptide products of *phnC*, *phnD* and *phnE* respectively are an ATP-binding subunit, a periplasmic binding protein and a Pn permease. The next gene, *phnF* encodes, based on sequence similarity, a negative regulatory protein that represses Pn transport via PhnCDE. The remaining ten genes of the operon (*phnGHIJKLMOP*) code for enzymes involved in Pn catabolism, such as a C-P lyase complex.

Owe to the presence of an 8 bp insertion in *phnEE. coli* K-12 strains do not express the PhnE permease and consequently are unable to take up phosphonates [5]. However, *phnE*^+^ revertants that arise by spontaneous slippage [5, 6] are relatively easy to select by growing bacteria with Pn as the sole phosphorus source or by growing bacteria in Pi-limited medium for several days [5–7]. The vast majority of *E. coli* strains and isolates carry a functional *phnE* gene.

*E. coli* possesses three Pi-transport systems - PstSCAB (Pst), PitA and PitB. Pst is an ABC-type high-affinity system formed by four proteins: the Pi-binding periplasmic protein PstS, the integral membrane proteins PstC and PstA, and the ATPase PstB. The *pst* operon belongs to the PHO regulon and responds to Pi-limitation. In addition to its role in Pi uptake, the Pst system also acts as a repressor of the PHO regulon under Pi-excess conditions [8].

PitA and PitB are metal-phosphate symporters that share 81% sequence identity. The former is likely constitutive, while PitB is inhibited by the PHO regulon [9] and is not functional under Pi-starvation or in a PHO-constitutive background, i.e., in a *pst* mutant [9–11].

Based on the ability to grow in media containing different phosphorus sources, Metcalf and Wanner [12] suggested that Phn is able to take up Pi, phosphite and Pi-esters. However, the bacterial strains used in that study were not fully characterized and the role of the PhnCDE system in Pi transport was not further confirmed.

By characterizing a spontaneous Phn^+^ revertant in *E. coli* K-12 carrying knockouts in genes related to Pi transport we unequivocally show that Phn has the ability to take up Pi in the absence of canonical Pi-transport systems and to support the growth of bacteria lacking another functional Pi-transport system. It is also shown that despite previous suggestions, the Ugp system is unable to transport Pi.

## Results

### Selection of a *phnE*^+^ revertant in *E. coli* K-12

PHO-constitutive mutants can be isolated by plating wild-type bacteria on glycerol-2-phosphate (G2P) as the sole C source. We have previously observed that the frequency of such mutants in bacteria carrying both *ΔpitA* and *Δugp* knockouts is extremely low [13]. This is because as most PHO-constitutive mutations occur in one of the five genes of the *pst* operon, a *ΔpitApst* strain would lack a functional Pi-uptake system and will thus not be able to grow with Pi as a phosphorus source. Furthermore, most G2P, which could in principle be utilized as a Pi source [14], is hydrolyzed to glycerol and Pi in the periplasm by the constitutively expressed AP, while the remaining intact G2P cannot be taken up due to the absence of Ugp or another G2P-transport system. Nevertheless, a small number of *ΔpitA* *Δugp* spontaneous *pst* mutants could be isolated on G2P, suggesting that the triple mutant acquired a fourth mutation that enabled growth in this medium.

To further investigate this matter, we attempted constructing a *ΔpitA* *Δugp* *Δpst* triple mutant in the wild-type K-12 strain MG1655 by means of recombineering and transduction. Individual deletions obtained by *λ*-*red* mediated recombineering were transduced one after another into MG1655 (see Methods). While all pairwise combinations could be easily obtained, attempts to originate the triple mutant were mostly unsuccessful. In one of these attempts, *Δpst* was transduced into the *ΔpitA* *Δugp* double mutant and the selective plate (L-agar containing kanamycin) was inadvertently kept at 37 ^∘^C for 72 h. A single colony emerged after 72 h which, once isolated was able to grow in LB and in minimal medium containing Pi as the sole P source. Genome sequencing of this bacterium (strain RI57) revealed the deletion of 8 bp (5’-GCTGGCGT) at position 407-414 of *phnE* ORF.

In addition to the three knockouts (*Δpst* *ΔpitA* *Δugp*) and the *phnE* 8 bp deletion, other point mutations in the genome of strain RI57 were observed (Table 1). One of them is a G insertion in *glpR* ORF, which encodes the repressor of the glycerol-3-phosphate regulon [15]. Some variants of strain MG1655 are *glpR*^-^ while others, like the one used in this study carry a wild-type copy of this gene [16]. With the exception of the 1 bp insertion in *glpR* none of these substitutions is apparently connected to the PHO regulon or to Pi metabolism. The 8 bp deletion in *phnE* was subsequently confirmed by Sanger sequencing.

**Table 1:** Mutations in strain RI57

**Gene/Region**	**Product**	**Genome position**	**Mutation type**	**AA change**
*lnt*	Apolipoprotein N-acyltransferase	689,456	SNP (G →C)	P35A
*shiA*	Shikimate:H ^+^symporter	2,054,570	SNP (G →A)	A310T
*glpR*	G3P repressor	3,560,455	INDEL (C →CG)	Cryptic gene in Ref. strain
*ugpBAEC*	G3P transporter	3,588,201-3,592,289	K0	Operon Knockout
*pitA*	Phosphate transporter	3,637,662-3,639,121	K0	Gene Knockout
*phnE*	Phosphonate transporter subunit	4,322,802	INDEL (GCGCCAGCA →G)	Reversion of frameshift
*pstSCAB-phoU*	Phosphate transporter	3,906,884-3,911495	K0	Operon Knockout
*idnT*	L-idonate/5-ketogluconate /gluconate transporter	4,491,280	SNP (A →T)	W416R

### PhnCDE supports growth with Pi in the absence of canonical Pi-transport systems

The ability of the triple knockout *ΔpitA* *Δpst* *ΔugpphnE*^+^ (strain RI57) to grow in LB medium that contains 2.2 mM Pi as the main phosphorus source [17] suggests that the PhnCDE system might be involved in the uptake of Pi in the absence of a viable Pi-transport system. To investigate this assumption a set of bacterial constructs was generated. First, the *phnE*^+^ allele was transduced from BL21, an *E. coli* B strain to MG1655 (strain RS03), then the *ΔpitA*, *Δugp* and *Δpst* deletions were sequentially transduced to MG1655 *phnE*^+^ originating strain RS07 (*phn*^+^3 *Δ*). In parallel, a *phnCDE* deletion was constructed and transferred to each one of the following double mutants: *Δugp* *Δpst*, *ΔpitA* *Δpst* and *ΔpitA* *Δugp* resulting in strains RS04 (*pitA*^+^3 *Δ* = *pitA*^+^ *Δpst* *Δugp* *Δphn*), RS05 (*ugp*^+^3 *Δ* = *ugp*^+^ *ΔpitA* *Δpst* *Δphn*) and RS06 (*pst*^+^3 *Δ* = *pst*^+^ *ΔpitA* *Δugp* *Δphn*), respectively.

**Fig. 1:**
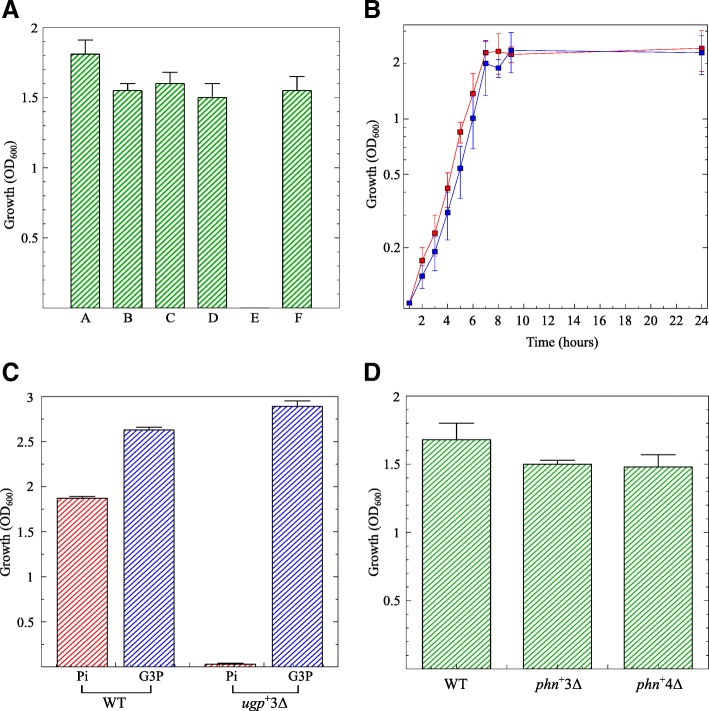
Growth of the triple knockouts in minimal medium. (**a**) Bacteria were grown overnight in TGP medium, with 1 mM Pi as a phosphorus source. **a**, wild-type strain MG1655; **b**, strain RI57 (*ΔpitA* *Δpst* *ΔugpphnE*^+^ - spontaneous *phnE*^+^ revertant); **c**, strain RS07 (*phnE*^+^3 *Δ*); **d**, strain RS04 (*pitA*^+^3 *Δ*); **e**, strain RS05 (*ugp*^+^3 *Δ*); **f**, strain RS06 (*pst*^+^3 *Δ*). (**b**) Growth curve of strain RS07 (*phnE*^+^ 3 *Δ*) in TGP. Bacteria were diluted to an OD _600_ of 0.1 and grown for 24 h in medium TGP (1 mM Pi). (red square), wild-type strain MG1655; (blue square), RS07 (*phnE*^+^3 *Δ*). (**c**) Growth of strain RS05 ()(*ugp*^+^ 3 *Δ*) with Pi or glycerol phosphate as phosphorus sources. Bacteria were grown overnight in TGP (1 mM Pi) or TGG3P (1 mM G3P). WT, wild-type strain MG1655. Red crossed stripes, TGP medium; blue cross stripes, TGG3P medium. (**d**) Growth of the triple and quadruple mutants RS07 (*phn*^+^3 *Δ*) and RS08 (*phn*^+^ *ΔpitA* *ΔpitB* *Δpst* *Δugp*) in TGP medium; WT, strain MG1655. Each bar represents the mean ± S.E.M. of three independent cultures

The new constructs were tested for overnight growth in minimal medium with 1 mM KH _2_*PO*_4_ as the sole phosphorus source (Fig. 1a). As expected, bacteria carrying either PitA or Pst grew almost as well as the wild-type strain. In the absence of both PitA and Pst, only the strains that possessed a functional PhnCDE system managed to grow. This includes the spontaneous *ΔpitA* *Δugp* *ΔpstphnE*^+^ revertant (strain RI57) and the *phnE*^+^3 *Δ* transductant (strain RS07). The *ugp*^+^3 *Δ* triple mutant (strain RS05) carrying a functional Ugp system did not grow in this medium.

To further investigate the ability of the PhnCDE system in supporting growth in the presence of Pi as the only phosphorus source, the *phn*^+^3 *Δ* triple knockout (strain RS07) and the wild-type strain MG1655 were set to grow in medium TGP (1 mM Pi) for 24 h (Fig. 1b). It can be observed that the growth rate of the *phn*^+^ triple mutant at the exponential phase (µ = 0.52 h^-1^) was as fast as that of the wild-type strain (µ = 0.46 h^-1^), suggesting that PhnCDE can take up Pi at a rate that enables maximal growth rate.

The only triple deletion that did not grow in medium TGP was strain RS05 (*ugp*^+^3 *Δ*), which suggests that the Ugp system is unable to transport Pi. To exclude the possibility that the *ugp* operon in this strain has acquired a spontaneous null mutation, the wild-type strain and the triple mutant *ugp*^+^3 *Δ* were grown in minimal medium supplemented with either Pi (TGP) or G3P (TGG3P) as phosphorus sources. Figure 1c shows that the growth yield of strain RS05 (*ugp*^+^3 *Δ*) in medium TGG3P was slightly higher than that of the wild-type strain, while in the TGP medium the *ugp*^+^3 *Δ* mutant grew very poorly. This indicates that strain RS05 (*ugp*^+^3 *Δ*) carries a functional Ugp system.

We also investigated whether the *pitB* gene, that encodes a PitA-like Pi-transporter that is normally nonfunctional in wild-type *E. coli* or in a PHO-constitutive mutant [9] was spontaneously activated enabling the growth of the *phn*^+^3 *Δ* triple mutant (strain RS07) in TGP. To test this possibility the *pitB* gene was deleted from strain *phn*^+^3 *Δ* resulting in the quadruple mutant *phn*^+^ *ΔpitA* *ΔpitB* *Δpst* *Δugp* (strain RS08 = *phn*^+^4 *Δ*). Figure 1d shows that both strain RS07 (*phn*^+^3 *Δ*) and RS08 (*phn*^+^4 *Δ*) grew in medium TGP, suggesting that PitB is not functional and confirming that the PhnCDE system is able to support growth with Pi as a phosphorus source.

### The PhnCDE system transports Pi

**Fig. 2:**
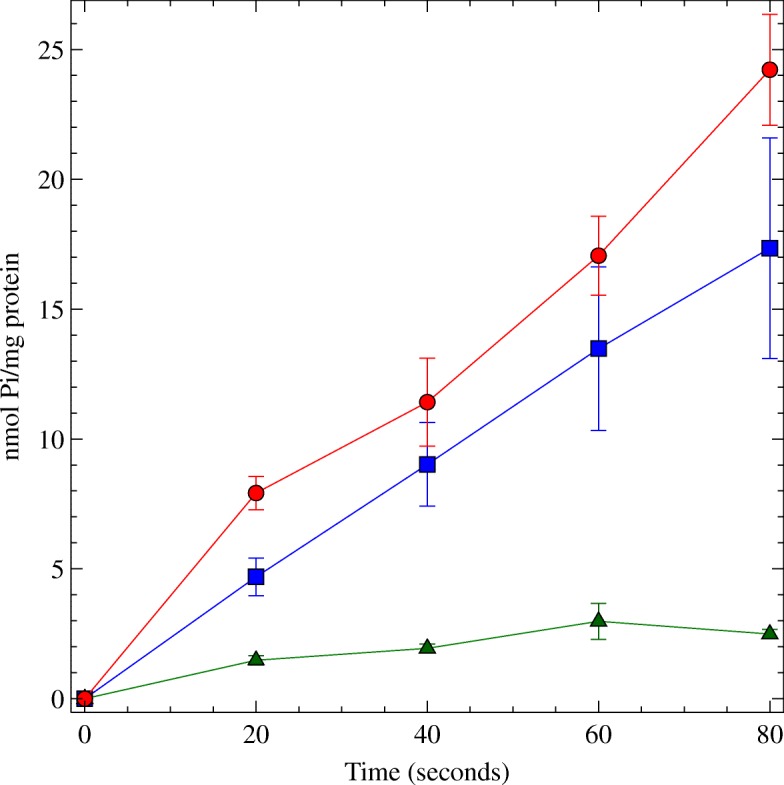
Pi uptake via PhnCDE and Ugp. Bacteria were resuspended in TG medium containing 0.2 mM ^32^P(KH _2_*PO*_4_). Samples were withdrawn every 20 s and the level of incorporated radioactivity (cpm counts) was measured. Blue square, strain RS07 (*phn*^+^3 *Δ*); green triangle, strain RS05 (*ugp*^+^3 *Δ*); red circle, wild-type strain. Each bar represents the mean ± S.E.M. of three independent cultures

Next, Pi uptake assays were performed in the wild-type strain MG1655, and in the triple knockouts *phn*^+^3 *Δ* (strain RS07) and *ugp*^+^3 *Δ* (strain RS05). Bacteria grown in medium TGP or TGG3P to an OD _600_ of 1.0 were washed to remove any phosphorus leftovers and exposed to 0.2 mM ^32^P(KH _2_*PO*_4_). Figure 2 shows that the rate of Pi uptake in strain RS07 (*phn*^+^3 *Δ*) was similar to that of the wild-type strain, while the *ugp*^+^3 *Δ* (strain RS05) failed to take up significant amounts of Pi. To further characterize the PhnCDE system the kinetic parameters of Pi uptake via Phn and PitA were obtained. The triple mutants *phn*^+^3 *Δ* (RS07) and *pitA*^+^3 *Δ* (RS04) were exposed to increasing concentrations of ^32^Pi and assayed for Piuptake. The kinetic parameters of Pi-uptake for strains RS07 (*phn*^+^3 *Δ*) and RS04 (*pitA*^+^3 *Δ*) were calculated from a Lineweaver-Burk double reciprocal plot (Additional file 1: Figure S1). The Vmax and Km of PhnCDE (strain RS07) were, respectively, 76.25 nmol Pi/min/mg protein and 19.46 µM Pi, while that of PitA (strain RS04) were 99.98 nmol Pi/min/mg protein and 25.99 µM Pi. Wilsky and Malamy [18] have reported for PitA a Vmax of 55 nmol Pi/min/mg protein and Km of 38.2 µM Pi. Altogether, these data indicate that the kinetics of the PhnCDE transport system towards Pi is very similar to that of the PitA transporter.

The uptake of Pi by PhnCDE was also validated by competing Pi with aminoethylphosphonate (AePn). Bacteria were exposed to 10 µM ^32^Pi and 50 s later an excess concentration of AePn (4 mM) was added. Additional file 1: Figure S2 shows that addition of AePn strongly inhibited Pi uptake when compared to the control which received no AePn. Lower concentrations of AePn resulted in less transport inhibition (data not shown).

Finally, we assayed Pi consumption throughout a bacterial growth curve. Strains MG1655 and RS07 (*phn*^+^3 *Δ*) were grown in medium TGP containing 1 mM Pi as the sole phosphorus source. At 1 h intervals, samples were taken for growth assessment (OD _600_) and residual Pi in the medium. Additional file 1: Figure S3 shows that the patterns of growth and Pi-consumption were very similar in both strains throughout the entire curve. In both cases, almost all Pi was exhausted after 24 h. These results indicate that Pi uptake via the PhnCDE system continues for several hours as efficiently as in the wild-type strain.

## Discussion

The data presented here show that in the absence of a canonical Pi-transport system, PhnCDE confers on bacteria the ability to take up Pi and to grow as fast as the wild-type strain. The functional redundancy of three different Pi-transport systems (PitA, Pst and Phn) attests for the importance of Pi as a vital macronutrient. However, it should be noticed that each one of the aforementioned transport systems play, at least one additional role or function. PitA is a symporter of Zn ^2+^ and other divalent cations [19] and Pst is a repressor of the PHO regulon [8]. Thus it is not surprising that Phn possesses more than one physiological role, namely transport of phosphonates, Pi and perhaps of other phosphorus-containing molecules [12].

The PhnCDE transport system in *E. coli* K-12 is normally cryptic, due to an 8 bp insertion in *phnE* ORF [5]. Deletion of the 8 bp insertion restores the reading frame and activates phosphonate transport via PhnCDE. Thus the 8 bp deletion observed in our *ΔpitA* *Δpst* *Δugp* transductant was likely to cause the reactivation of PhnCDE. Selection of *phnE*^+^ revertants has been reported before in bacteria plated on minimal medium with phosphonate as the sole phosphorus source [5, 6] and by serial transfer of a wild-type strain growing in low Pi minimal medium [7, 20].

Of the four tested transport systems, only Ugp was unable to support growth and take up Pi. In a previous study, we have shown that the emergence of PHO-constitutive mutants on plates containing glycerol-2-phosphate (G2P) as the sole carbon source partially depends on the presence of Ugp [13]. The hypotheses raised to explain this phenomenon were that Ugp was required for the uptake of either G2P or Pi. The finding that Ugp is unable to take up Pi indicates that the contribution of Ugp to the emergence of PHO-constitutive mutants is likely to be associated with the uptake of G2P [14]. It should be noted, however, that the ability of Ugp in transporting G2P is still unsettled, as genetic evidences showed that Ugp transports G2P [14], while in vitro, UgpB, the periplasmic binding protein, did not bind this molecule [21].

An early report suggested that *E. coli*’s PhnCDE is able to take up Pi [12]. This assertion was based on the growth of strain BW4794, which when plated on phosphite or phosphonate acquired the ability to grow on both phosphorus sources and also on Pi. However BW4794 was a poorly defined mutant that carried a partial *pst* deletion and another undefined linked mutation. The fact that this strain was not clearly shown to be *pitA*-negative adds uncertainty to the identity of the transport system used for Pi uptake. To the best of our knowledge, since that publication the ability of the PhnCDE system of *E. coli* to transport Pi was not further tested and confirmed. There were, however, some hints that the PhnCDE system might be involved in Pi transport. Rizk et al. [22] reported that *E. coli* PhnD (the periplasmic binding protein) displays a relatively small affinity towards Pi (Kd = 50 µM). The PhnDCE system of *Mycobacterium smegmatis* was shown to transport Pi but not phosphonates [23] and the PhoCDET Pi transport system of *Sinorhizobium meliloti* transports both Pi and phosphonates [24].

## Conclusions

By sequentially deleting all known Pi transport-related genes we clearly demonstrated that Phn takes up Pi in sufficient amounts to support growth with Pi as the sole phosphorus source. It was also shown that reversion of the *phnE* mutation could be selected by plating the triple knockout *ΔpitA* *Δpst* *Δugp* on rich media, such as L-agar. This medium contains 2.2 mM free Pi [17] and possibly limiting concentrations of organic phosphates. Under these conditions, the triple knockout *ΔpitA* *Δpst* *Δugp* cannot grow unless by reverting the *phnE* insertion.

## Methods

### Bacterial strains and growth conditions

Bacterial strains used in this study are depicted in Table 2. Unless otherwise specified, bacteria were incubated at 37 ^∘^C under aerobic conditions. LB/L-agar is the standard rich medium [25]. Minimal medium was composed by T-salts (0.12 M Tris-HCl, 80 mM NaCl, 20 mM KCl, 20 mM NH_4_Cl, 0.98 mM MgCl_2_.6H_2_O, 2.46 mM Na_2_SO_4_, 2 mM CaCl_2_, 2 µM FeCl_3_, 2 µM ZnCl_2_, pH 7.5) supplemented with glucose (11 mM) and 1 mM of the following P sources: KH_2_PO_4_ (TGP) or a glycerol phosphate isomeric mixture (glycerol-3-phosphate + glycerol-2-phosphate) (TGG3P) [26].

**Table 2:** Strains and oligonucleotides used in this study

**Strain**	**Relevant phenotype or sequence**	**Reference**
**MG1655 derivatives**		
MG1655	*E. coli* K-12 *phnE*	Lab collection
RI57	MG1655 *ΔpitA* *ΔugpBAEC* *ΔpstSCAB-phoUphnE*^+^ (spontaneous *phnE*^+^ revertant)	This Study
RS02	MG1655 *ΔphnCDE*::Km	This Study
RS03	MG1655 *phnE*^+^ (P1 transduction from BL21 to MG1655)	This study
RS04 (*pitA*^+^3 *Δ*)	MG1655 *ΔphnCDE* *ΔugpBAEC* *ΔpstSCAB-phoU* (*pitA*^+^)	This Study
RS05 (*ugp*^+^3 *Δ*)	MG1655 *ΔphnCDE* *ΔpitA* *ΔpstSCAB-phoU* (*ugpBAEC*^+^)	This Study
RS06 (*pst*^+^3 *Δ*)	MG1655 *ΔpitA* *ΔphnCDE* *ΔugpBAEC* (*pstSCAB-phoU*^+^)	This Study
RS07 (*phn*^+^3 *Δ*)	MG1655 *ΔpitA* *ΔugpBAEC* *ΔpstSCAB-phoU* (*phnE*^+^)	This Study
RS08 (*phn*^+^4 *Δ*)	MG1655 *ΔpitA* *ΔpitB* *ΔugpBAEC* *ΔpstSCAB-phoU* (*phnE*^+^)	This Study
**Other Strains**		
BL21	*E. coli* B *phnE*^+^	
KM32	*ΔrecBCD*::P*tac-gam-bet-exo* cat	[28]
KM44	*ΔrecBCD*::P*tac-gam-bet-exo* kan	[28]
Oligonucleotides		
pitA_mut_Fow	TCAAAATGGCGTAACGTCCTATGCTACATTTGTTTGCTGGGTGTAGGCTGGAGCTGCTTC	
pitA_mut_Rev	GCTCGTTTTGGTGCGTACGATTACAGGAACTGCAAGGAGACATATGAATATCCTCCTTAG	
pitA_ver_Fow	TTGTAAAGATTCCTCAGTGGTC	
pitA_ver_Rev	TGATGCGCTACGCTTATCAG	
pitB_mut_Fow	GAAATGCCCGATCGCCAGGACCGGGCATTTTCAGGAAGGGTGTAGGCTGGAGCTGCTTC	
pitB_mut_Rev	TGCGTCCGTTCGTAAATTCAAAATGGCGTAATCTAATATCCATATGAATATCCTCCTTA	
pitB_ver_Fow	TTAACCAGTGGAATACCTGTG	
pitB_ver_Rev	CGAGGAAGATTGCCATAACG	
phn_mut_Fow	GGATGTGTAGACAAGTGCATAGATATCAATGCCTCGCTTAGTGTAGGCTGGAGCTGCTTC	
phn_mut_Rev	ATCCGCCACGATGGAGCCACTTTTTTAGGGAGGCTGCATCCATATGAATATCCTCCTTAG	
phn_ver_Fow	TTTGCGGCTATCTCTTGATAGC	
phn_ver_Rev	GTCACAATAATCCGCCACGA	
phn_seq_Fow	GTCACAATAATCCGCCACGA	
pst_mut_Fow	GTCTGGTGAATTATTTGTCGCTATCTTTCCCCGCCAGCAGTGTGTAGGCTGGAGCTGCTTC	
pst_mut_Rev	AGGAGACATTATGAAAGTTATGCGTACCACCGTCGCAACTCATATGAATATCCTCCTTAG	
pstS_S_Fow:	TGTAATTGACTGAATATCAACGCT	
phoU_S_Rev:	GCTCGCAGTTATTAACTTTGTG	
ugp350	AACGATGAAACCGTTACATTATACAGCTTCAGCACTGGCGGTGTAGGCTGGAGCTGCTTC	
ugp4480	CCAGATGCAGCCACAGCGTGCTGCCTGCCGTCGGGCGCTCCATATGAATATCCTCCTTAG	
ugp858	ACCGCCTTGTCATCTTTCTG	
ugp5210	CTCGTTGTCCTGTTTCACC	

### Gene and operon knockouts

Gene and operons were deleted using the *λ*-*red* recombinase system as originally described by Datsenko and Wanner [27] and Murphy et al. [28]. Briefly, the *cat* or *kan* resistance cassettes were amplified from plasmids pKD4 or pKD3, respectively, using the following pairs of DNA oligos: ugp350 and ugp4480 for deletion of *ugpBAEC*; pitA_mut_Fow and pitA_mut_Rev for deletion of *pitA*; pitB_mut_Fow and pitB_mut_Rev for deletion of *pitB*; pst_mut_Fow and pst_mut_Rev for deletion of the *pstSCAB-phoU* operon; and phn_mut_Fow and phn_mut_Fow for deletion of the *phnCDE* genes. The amplicons containing the *cat* or *kan* cassettes were electrotransformed in strains KM44 or KM32. To induce the *λ*-red genes the bacterial cultures were supplemented with 1 mM of IPTG. Selection of recombinants was done by plating on L-agar supplemented with chloramphenicol or kanamycin. Knockouts of *pitA*, *pitB*, *phn*, *pst* and *ugp* were confirmed by PCR using primers pitA_ver_Forw/pitA_ver_Rev, pitB_ver_Forw/pitB_ver_Rev, phn_ver_Forw/phn_ver_Rev, pstS_S_Fow/phoU_S_Rev and ugp858/ugp52101 and, when applicable, by phenotypic assays, such as AP activity [29] or growth on phosphonates. The deletions were transferred to strain MG1655 by P1 transduction. The antibiotic resistance marker (*kan* or *cat*) was eliminated from the recombinant chromosomes by transformation with the temperature-sensitive plasmid pCP20 as described [27].

### P1 transduction

Transfer of chromosomal markers using P1 transduction was performed as described [25]. Knockout transductants were selected on L-agar plates containing either kanamycin or chloramphenicol. Selection of the *phnE*^+^ allele transductant (from strain BL21 to MG1655) was in minimal medium plates containing 2-aminoethyl-phosphonic acid as a phosphorus source.

### Genome sequencing

Genomic DNA from strain RI57 was extracted using the Wizard Genomic DNA purification kit (Promega) following the manufacturer instructions. Quantification of DNA as well as sample integrity and purity was assessed using a QuBit dsDNA BR kit (Thermo Fisher) as specified by the manufacturer. For library construction, DNA was fragmented in a Covaris ultrasonicator and library preparation was performed as specified by Illumina Mi-Seq protocols. Sequencing was performed in an Illumina Mi-seq sequencer at the Centro de Facilidades de Apoio a Pesquisa (CEFAP-USP). Genome sequence was assembled and annotated using as reference the genome sequence of strain MG1655 (gi = 556503834 ref = NC_000913.3). The draft genome sequence of strain RI57 was deposited at DDBJ/EMBL/GenBank under the accession number CP032679.

### Sanger sequencing

Local sequencing was performed by the Centro de Estudos do Genoma Humano (CEGH) at the Universidade de São Paulo. Amplicons produced by PCR using oligos phn_seq_Fow and phn_seq_Rev were purified using a Wizard PCR purification kit (Promega) and DNA sequencing was performed by the Sanger method using the BigDye terminator version 3.1 Cycle Sequencing Kit (Applied Biosystems) and analyzed in a ABI 3730 DNA analyser.

### Pi uptake assay

Bacteria were grown to the early exponential phase (OD ∼0.15), washed with 0.9% NaCl and resuspended in the same volume of TG medium (TGP lacking KH _2_*PO*_4_). For the Pi uptake assays of MG1655, *phn*^+^3 *Δ* and *ugp*^+^3 *Δ* a solution of 200 µM KH _2_*PO*_4_ mixed with 1 µCi ^32^P was used. To obtain the kinetic parameters of Pi-uptake of strain RS07 (*phn*^+^3 *Δ*) and RS04 (*pitA*^+^3 *Δ*) KH _2_*PO*_4_ solutions of 5, 7.5, 10, 15 and 20 µM and 15, 20, 30 and 50 µM were, respectively, prepared. The “cold” KH _2_*PO*_4_ solutions were mixed with 1 µCi ^32^P. The ^32^P(KH _2_*PO*_4_) solutions were used to start the uptake assay. At several intervals after the addition of ^32^P(KH _2_*PO*_4_), 500 µl of the bacterial cultures were withdrawn, filtered under vacuum through a cellulose acetate filter (0.22 µm) and washed with 5 ml of a TP solution (T-salts + 10 mM KH _2_*PO*_4_) to remove the unincorporated ^32^Pi. The filters were then move to vials filled with scintillation cocktail (Optiphase Hisafe 3, Perkin Elmer) and the radioactive content of each sample was measured in a *β*-counter.

### Growth curves and determination of Pi

The wild-type strain MG1655 and strain RS07 (*phn*^+^3 *Δ*) were grown in medium TGP containing 1 mM KH _2_*PO*_4_. Samples were taken hourly for assessing the OD_600_ and residual Pi in the medium. Pi concentration in the culture medium was measured as described [30]. The supernatants of the bacterial cultures were collected, diluted 100 times and mixed with the same volume of the working reagent (1 volume of 167 mM H _2_*SO*_4_, 1 volume of 2.5% (NH _4_) _2_*MoO*_4_, 1 volume of 10% ascorbic acid and 2 volumes of deionized water) and incubated at 37 ^∘^C for two hours. Following the incubation period, samples were measured in a spectrophotometer at 820 nm. Calibration solutions were prepared with the following concentrations of NaH _2_*PO*_4_: 0.16 mM, 0.08 mM, 0.04 mM, 0.02 mM and 0.01 mM.

## Additional file

Additional file 1 A supplement containing **Figures S1-S3.** (pdf 150 kb)

## Abbreviations

AePn: Aminoethylphosphonate
AP: Alkaline phosphatase
Cat: Chloramphenicol resistance marker G2P: Glycerol-2-phosphate
G3P: Glycerol-3-phosphate
µ: Growth rate
kan: Kanamycin resistance marker
LB: Lysogeny broth
OD: Optical density
Pi: Phosphate
Pn: Phosphonate
TGG3P: Minimal medium containing glycerol-3-phosphate as a P source
TGP: Minimal medium containing phosphate as a P source
