# Supplementary material for: Phosphate uptake by the phosphonate transport system PhnCDE
Source: BMC Microbiol. 2019 Apr 16;19:79. doi: 10.1186/s12866-019-1445-3 (PMC6469041; doi:10.1186/s12866-019-1445-3)
Supplement: Supplementary file 1 — A supplement containing Figures S1-S3. (pdf 150 kb) [file 12866_2019_1445_MOESM1_ESM.pdf]

Supplemental Material

Phosphate uptake by the phosphonate transport system PhnCDE

Raffaele Stasi[1], Henrique Iglesias Neves[1], and Beny Spira[1]*

[1]Departamento de Microbiologia, Instituto de Ciências Biomédicas Universidade de São Paulo, São Paulo-SP, Brazil

*Corresponding author, email: benys@usp.br, phone: 5511 30918346

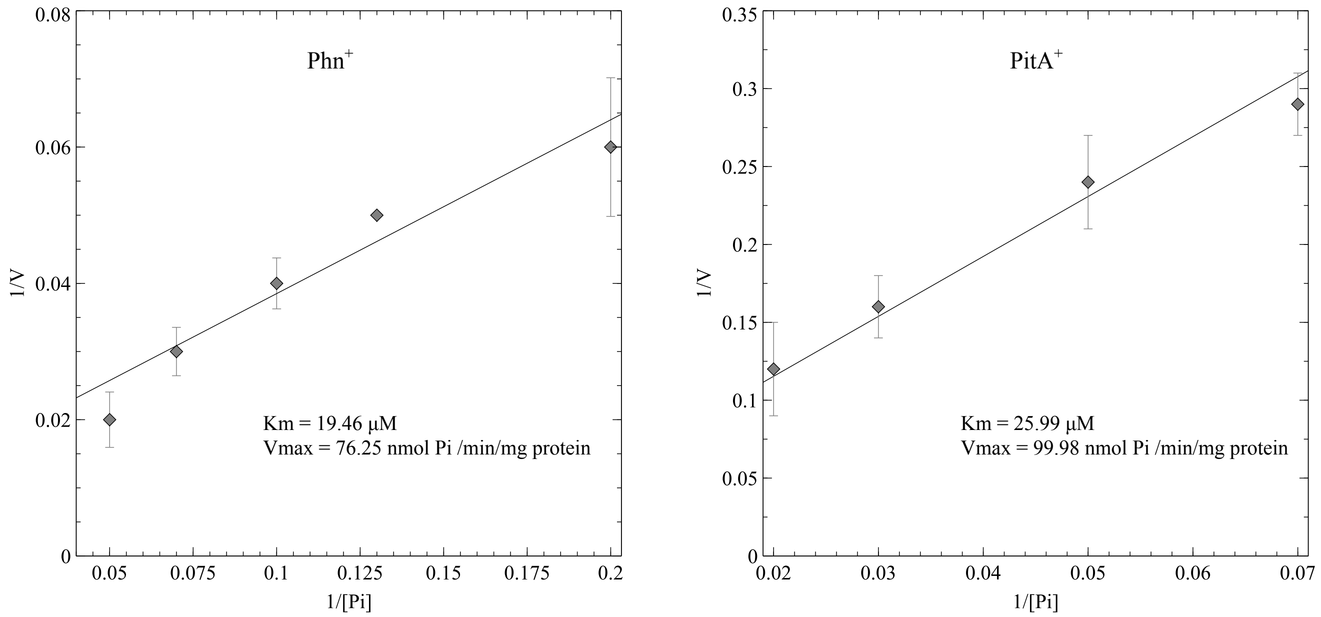

Fig S1. Lineweaver-Burk plots of ^{32}Pi uptake by strains *phn*$^{+}$3Δ and *pitA*$^{+}$3Δ. Bacteria were suspended in TG medium containing 5 µM to 20 µM ^{32}Pi in the case of *phn*$^{+}$3Δ and 15 µM to 50 µM ^{32}Pi in the case of *pitA*$^{+}$3Δ, at which point samples were withdrawn every 10 seconds. The V_0 obtained with each Pi concentration were used to plot the kinetics of Pi uptake and to draw the K_m and V_{max} towards Pi of the transport systems. Each point represents the mean ±SEM of at least three independent assays.

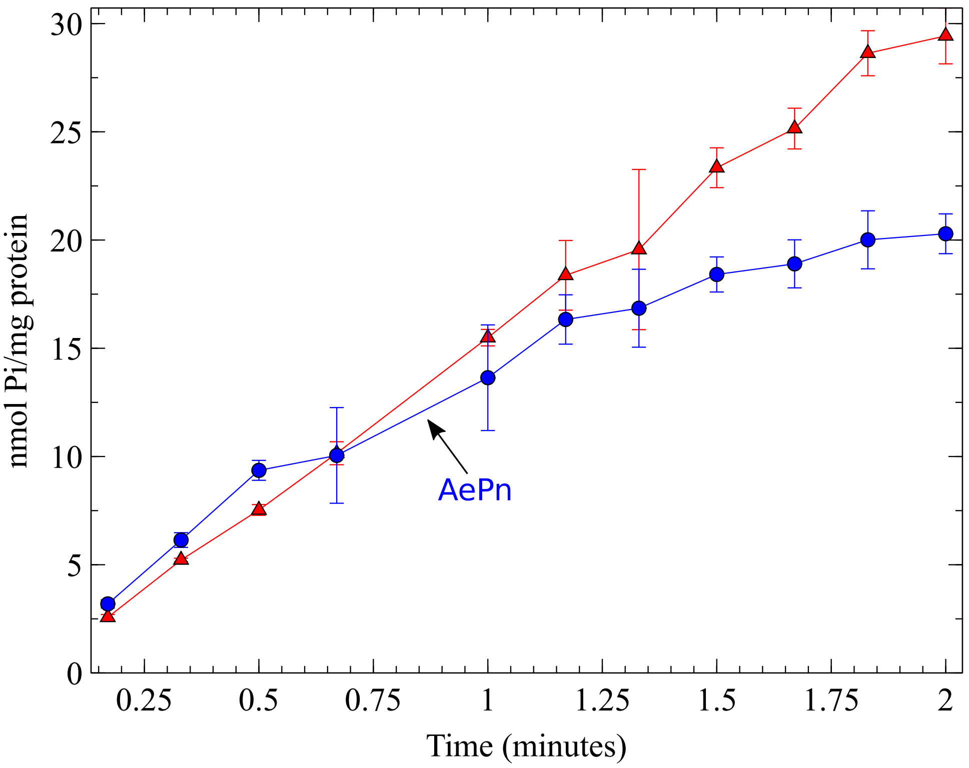

Fig S2. Inhibition of Pi-uptake by AePn. Two cultures of the *phn*$^{+}$3Δ strain was suspended in medium TGP (10 µM ^{32}Pi). Samples were withdrawn every 10 seconds and the level of incorporated radioactivity (cpm counts) was measured. At 50 s, 4 mM AePn were added to one of the cultures. Each point represents the mean ±SEM of three independent assays.

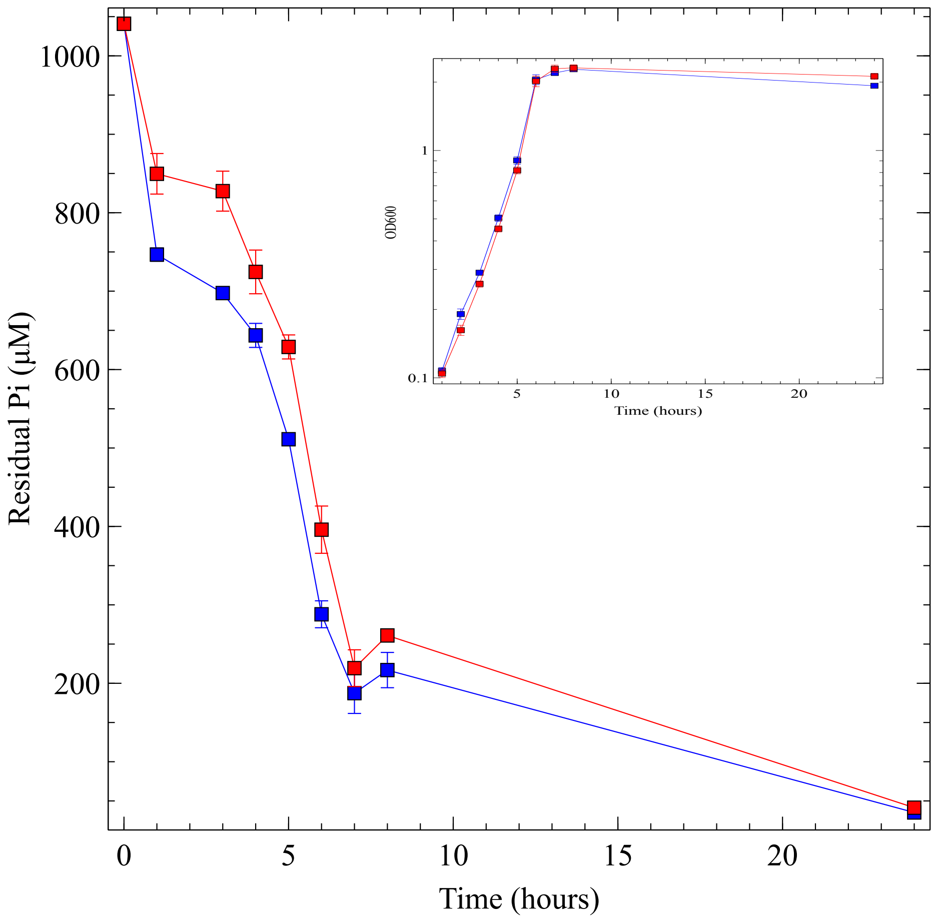

Fig S3. Pi consumption by strains *phn*$^{+}$3Δ(RS07) and MG1655. Bacteria were grown in medium TGP (1 mM KH_2PO_4). At the depicted time intervals samples were analysed for residual Pi in the medium and growth (OD_{600}) (inset). Each point represents the mean ±SEM of three independent assays.
